# Effect of the Synthesis Conditions of MIL-100(Fe) on Its Catalytic Properties and Stability under Reaction Conditions

**DOI:** 10.3390/ma15186499

**Published:** 2022-09-19

**Authors:** José J. Delgado-Marín, Javier Narciso, Enrique V. Ramos-Fernández

**Affiliations:** 1 Laboratorio de Materiales Avanzados, Departamento de Química Inorgánica, Instituto Universitario de Materiales de Alicante, Universidad de Alicante, Apartado 99, 03080 Alicante, Spain; 2 Instituto de Investigación Sanitaria y Biomédica de Alicante (ISABIAL), 03690 Alicante, Spain

**Keywords:** metal–organic framework, catalysts, stability

## Abstract

MIL-100(Fe) is a metal–organic framework (MOF) characterized by the presence of Lewis acid and Fe(II/III) redox sites. In this work, different synthesis methods for the preparation of MIL-100(Fe) are studied. Depending on the source of fluorine, different phases can be obtained: MIL-100(Fe) and an Fe trimesate with unknown structure which we call Fe(BTC). These materials were characterized using numerous techniques and applied in the reaction of CO_2_ cycloaddition with epichlorohydrin, a reaction catalyzed by Lewis acid sites. It was observed that samples with more Fe(BTC) phase were more active in the reaction. However, all samples, under reaction conditions, transformed into a less active phase.

## 1. Introduction

MOFs have aroused great interest within the scientific community, since they have great potential for various applications such as catalysis [1,2,3,4,5], sensing [6,7,8,9], or adsorption [10,11,12,13,14,15,16,17,18,19].

Among the wide variety of catalytic applications of MOFs, there are a number of families that are especially interesting to be used as catalysts. The UiO-66 family or the MIL-100-1 family are of great importance since they have the so-called open metal sites (OMS) and, in principle, they are very stable [20,21,22]. The OMS are metal coordination sites that are not coordinated with the linker forming the structure, and, when the molecules that coordinate these sites (H_2_O, solvent, –OH, –F) are removed, these sites are exposed. These sites are electron-deficient, i.e., they are Lewis acid sites. How to generate these sites and their acidic nature has been extensively studied [23].

Focusing on the MIL-100 and MIL-101 family, they are structures with mesoporous cavities accessible through microporous windows, which makes them especially interesting for catalytic applications since the diffusion of products and reagents through its structure is favored. The MIL-100 structure is made up of trimesic acid and different metals (V, Fe, Cr, Al, or Sc), while the MIL-101 structure is consists of the same metals, with the difference being that terephthalic acid is used as a linker. Within the different structures, MIL-100 is the most used in the cycloaddition of CO_2_ due to its large number of Lewis acid sites. In the field of catalysis, it is important to point out that MIL-100 made of Fe is of special interest since, in addition to having Lewis acidity, it can also have a redox functionality (Fe(III)/Fe(II)) [24,25,26,27,28,29,30,31,32,33,34,35,36,37,38,39]. Further, iron is cheap, nontoxic, and environmentally friendly compared with other metals.

BASF markets an iron trimesate Fe(BTC) that has a chemical composition very similar to MIL-100(Fe) and a high specific surface area, called F300. However, it is not known exactly if it is a crystalline material or an amorphous material since the diffraction patterns of the powder sample do not show peaks. This occurs either because Fe(BTC) material is made of very small crystals or because it is an amorphous material. Garcia et al. compared the catalyst performance of carefully prepared MIL-100(Fe) with that of Fe(BTC) marketed by BASF [40]. They have found that, while the commercial Fe(BTC) is the best catalyst for Lewis acid reactions because of its additional Brönsted acid sites, MIL-100(Fe) would be the best choice for oxidation reactions due to the presence of Fe^3+^/Fe^2+^ pairs. The presence of Brönsted acid sites in Fe(BTC) is due to the large number of defects in the structure. This shows that the quality of the material obtained after synthesis is important. Highly crystalline materials are not always the best options to be used as catalysts. In many occasions, defected MOF catalyzed the reaction better than crystalline phases.

To understand the physicochemical and catalytic properties of this fascinating material, it is necessary to know and finely control the synthesis parameters to know if pure MIL-100(Fe), mixtures of MIL-100(Fe) and Fe(BTC), or defected MIL-100(Fe) is obtained. It is known that the metallic clusters of this material are stabilized in the presence of –F, –Cl, or –OH groups. These species are introduced into the cluster during synthesis [41,42,43].

By controlling the source of F from which the MOF is synthesized, we can control the purity of the MOF structure generated. Therefore, it is expected that, by using HF, a purer MIL-100(Fe) will be generated, and, if we do not use HF in the synthesis, MIL-100(Fe) will not be generated or it will be very defective and not very crystalline if generated.

In this work, we aimed to prepare MIL-100(Fe) with different sources of F to understand the kinds of phases produced. In this way, we generate materials with different compositions and crystallinity to understand what effect they have on the performance of these catalysts in the CO_2_ cycloaddition reaction in epoxides [42,43].

In this contribution, we use different fluorine sources to prepare MIL-100(Fe), in order to be able to control the crystallinity and purity of MIL-100(Fe), as well as the formation of other phases. The materials were then characterized using a wide variety of techniques to analyze their properties. In addition, we used them as catalysts for CO_2_ cycloaddition with epoxides. We analyzed their performance as catalysts, as well as the influence of defects on their stability under the reaction conditions.

## 2. Materials and Methods

Different types of MIL-100(Fe) were prepared using the procedures described below. All samples were prepared in a similar way. The main difference was in the source of mineralizing agent used. We prepared a sample without a mineralizing agent and others using HF, KF, and a mixture of HF:KF.

The synthesis of **MIL-100(Fe)-NO** was carried out by dissolving iron nitrate nonahydrate (6.6 mmol) and 1,3,5-benzenetricarboxylic acid (4.4 mmol) in deionized water (75 mL). According to the structure of MIL-100(Fe), the molar ratio between iron and linker was 1:2/3. Afterward, the solution was heated in a Teflon-lined autoclave (100 mL volume) at 150 °C, keeping this temperature for 2 weeks. After this time, the autoclave was cooled over 3 h to room temperature. The resulting orange powder was washed in deionized water for 3 h and ethanol at 70 °C for 3 h. Then, the powder as dried at 80 °C overnight.

**For MIL-100(Fe)-HF,** ferric nitrate nonahydrate (6.6 mmol) and 1,3,5-benzenetricarboxylic acid (4.4 mmol) were mixed in aqueous solution of HF (6.6 mmol) and deionized water (75 mL). In accordance with the cluster, the molar ratio between iron and fluorine was 1:1/3, but a ratio of 1:1 was studied to determine if the structure was capable of assimilating more fluorine. The mixture was heated in a Teflon-lined autoclave (100 mL volume) at 150 °C for 2 weeks. After the autoclave was cooled over 3 h to room temperature, the solid was washed in deionized water and ethanol at 70 °C for 3 h. Then, the powder was dried at 80 °C overnight.

The reaction to synthesize **MIL-100(Fe)-KF** was prepared by heating iron nitrate nonahydrate (6.6 mmol), trimesic acid (4.4 mmol), and potassium fluoride (6.6 mmol) in deionized water (75 mL) in a Teflon-lined autoclave (100 mL volume) at 150 °C for 2 weeks. After cooling, the resulting orange solid was washed in deionized water and ethanol at 70 °C for 3 h to purify MIL-100 KF. Then, the MOF was recovered by centrifugation and dried.

**MIL-100(Fe)-HF:KF** was synthesized by adding iron nitrate nonahydrate (6.6 mmol), 1,3,5-benzenetricarboxylic acid (4.4 mmol), potassium fluoride (3.3 mmol), and an aqueous solution of HF (3.3 mmol) to deionized water (75 mL). In this case, to guarantee the necessary amount of fluorine, both HF and KF were administered at 3.3 mmol. The mixture was introduced in a Teflon-lined autoclave (100 mL volume) at 150 °C for 2 weeks. When the time was finished, the autoclave was cooled over 3 h to room temperature. A procedure of washing with deionized water and ethanol at 70 °C was carried out with the solid. Finally, the MOF was dried at 80 °C overnight. The porosity and specific surface area of the samples were characterized by means of nitrogen adsorption–desorption isotherms. The samples were outgassed at 150 °C for 10 h before the adsorption measurements. The analysis was measured at −196 °C in a Quadrawin (Quantachrome, Boynton Beach, FL, USA) device. The specific surface area was determined from the N_2_ adsorption branch and calculated using the Brunauer–Emmett–Teller method. In all cases, the number of points used to apply the BET equation was higher than 5, and the value of C was always positive. V_micro_ was estimated using the Dubinin–Raduskevich method.

X-ray diffraction analysis was used to identify crystallographic phases of the samples. The PXRD was recorded on Panalytical Empyrean diffractometer with a goniometer that had an X-ray tube (Kα, λ = 1.54 Å), fitted with a Cu cathode and a detector PIXcel 3D. The range of the spectra was registered between 0.5° and 70° with a step size of 0.01° and a step time of 20 s.

To study the leaching of Fe during the reaction, inductively coupled plasma mass spectrometry was used. The analysis was obtained in ICP-MS 8900 Agilent equipment. For the preparation of the samples, 100 µL of the final reaction product mixture was diluted in 5 mL of distilled water and shaken to mix homogeneously. The ICP was calibrated with standard solutions.

Thermogravimetric–mass spectroscopy (TG–MS) curves were registered on a TGA/STA 449 F5 Jupiter equipment coupled to a quadrupole mass spectrometer Aeolos QMS 403 Quadro, both from NETZSCH (Selb, Germany). The samples were exposed to a temperature range of 25–1000 °C with increasing temperature (10 °C·min^−1^) while the argon flow rate was held constant (50 mL·min^−1^).

The X-ray photoelectron spectroscopy study was carried out in a Kα spectrometer, from Thermo-Scientific. A rigorous deconvolution of the peaks was performed, and the peak areas were estimated by calculating the integral of each peak. The Shirley background was subtracted, and the experimental peak was fitted to a combination of Lorentzian/Gaussian with a ratio 30/70.

The Fourier-transform infrared (FT-IR) spectra were obtained using a Jasco FT-IR 4700 (detector DLaTGS) in ATR mode with a diamond crystal for the measurement of solid samples. The spectral range was from 900 to 4000 cm^−1^, monitored with a resolution of 1 cm^−1^. To study the spectra at 150 °C, a praying mantis diffuse reflectance accessory, from Harrick Scientific, was coupled to the FT-IR equipment. The chamber of samples was purged with helium to avoid the interference in the spectra with humidity or oxygen.

**Scheme 1:**
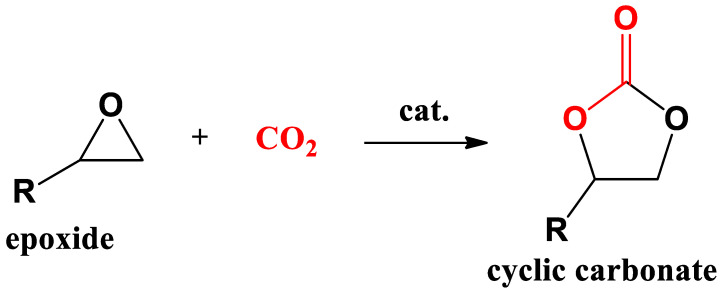
Synthesis of cyclic carbonates from an epoxide.

For the reaction to take place, MIL-100 was previously activated by heating at 150 °C overnight to remove the guest molecules such as water or ethanol. To carry out the reaction, epichlorohydrin (97 mol.%), tetramethylammonium bromide or tetrabuthylammonium iodide as cocatalyst (1.53 mol.%), mesitylene as NMR internal standard (0.57 mol.%), and the activated MIL-100(Fe) as catalyst (0.84 mol.%) were introduced in a steel vessel with a magnetic stirrer. After that, the reactor was closed and purged with CO_2_, before finally pressurizing to 15 bar and heating to 60 °C at a rate of 2 °C·min^−1^. During the heating process, the speed of stirring was low (200–300 rpm) until the temperature was reached in the vessel. Here, the speed of stirring was increased to 1000 rpm to start the reaction. The reactor was equipped with a system to extract small quantities of reaction mixture. The reaction mixture was separated from the catalyst using 0.2 µm nylon filters. NMR samples were prepared with 50 µL of reaction mixture and 600 µL of CDCl_3_, containing 0.03% (*v*/*v*) of trimethylsilane (TMS) as an internal standard. The analysis was performed using ^1^H-NMR spectroscopy on a 300 MHz Bruker spectrometer. Scheme 1 outlines the reaction that was performed.

## 3. Results and Discussion

**Figure 1:**
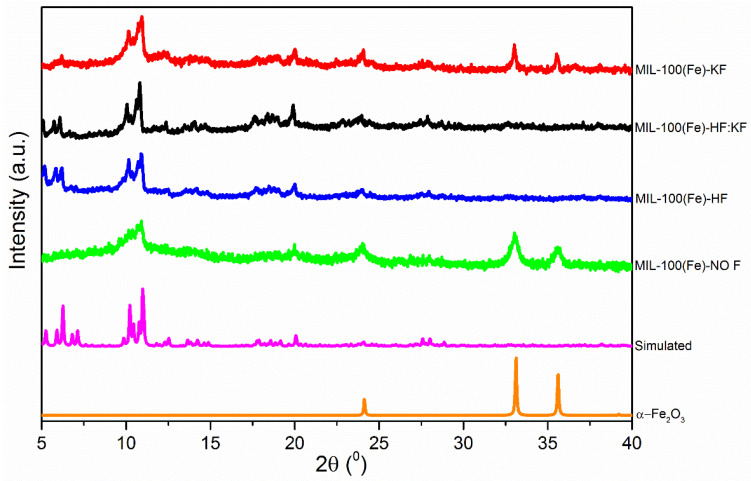
XRD pattern of the synthesized MIL-100(Fe) and simulated XRD pattern of MIL-100(Fe) (magenta) and α-Fe_2_O_3_.

The X-ray diffraction patterns and the analysis of these results are shown in Figure 1 and Table A1, respectively. The samples prepared with HF or HF:KF showed a better-defined diffraction pattern than those prepared without HF. The sample prepared with KF showed the MIL-100(Fe) peaks between 10° and 12.5°; however, the peaks between 5° and 7.5° were not present. These results are similar to those found by Dhakshinamoorthy [40], when he compared MIL-100(Fe) and commercial Fe trimesate (Fe(BTC)). The diffraction pattern of Fe(BTC) showed a broad peak between 10° and 12° 2*θ* and no peaks between 5° and 7.5° 2*θ*. These results indicate that we probably generated a mixture of known MIL-100(Fe) and unknown Fe(BTC) phases. In addition, we observed two peaks at larger 2*θ* which were ascribed to the α-Fe_2_O_3_ phase. This indicates that KF is not as good a mineralizing agent as HF and causes α-Fe_2_O_3_ to form.

With the X-ray diffraction results in hand, we can say that the presence of HF is essential to produce MIL-100(Fe) and that other crystalline phases such as α-Fe_2_O_3_ do not show up. However, we cannot know if there are any other amorphous phases that may then have an effect on its final application. To identify these possible amorphous phases, all samples were characterized by thermogravimetry analysis (TGA). The exhaust gases from these tests were analyzed by mass spectrometry. This is a powerful tool to know not only the stability of MOFs, but also the mechanism of decomposition.

**Figure 2:**
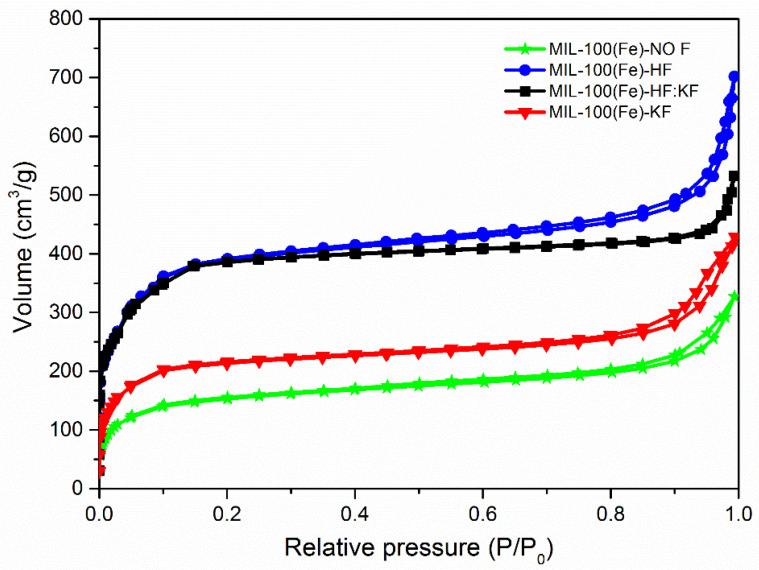
N_2_ adsorption–desorption isotherms of MIL-100(Fe) synthesized.

To understand the effect of the addition of fluorine to the synthesis mixture, we then characterized the prepared materials by means of nitrogen adsorption isotherms at −196 °C. Figure 2 shows these results. The four samples showed a type I isotherm according to IUPAC, characteristic of MIL-100(Fe). As can be seen, the sample prepared with HF (MIL-100(Fe)-HF) had the highest surface area, followed by the sample prepared with the HF:KF mixture. This shows that the presence of HF resulted in materials with higher porosity. It is known that fluorine helps to stabilize the cluster and, consequently, the crystallization of the MOF [44]. The sample prepared with a different source of fluorine (MIL-100(Fe)-KF) presented a lower specific surface area than that prepared with HF, but greater than that prepared in the absence of fluorine, indicating that not only is the presence of fluorine important, but also its source. The values for surface area, as well as micro- and mesopore volume, are shown in Table 1. The materials were essentially microporous with a surface area ranging from 453 to 962 m^2^/g. These values are slightly lower than those found in the literature.

**Figure 3:**
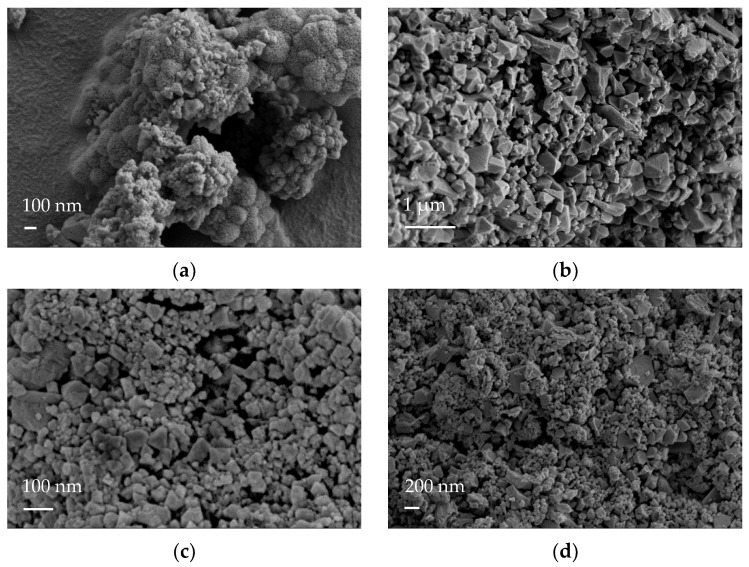
SEM micrographs of the samples: (**a**) MIL-100(Fe)-NO F; (**b**) MIL-100(Fe)-HF; (**c**) MIL-100(Fe)-HF:KF; (**d**) MIL-100(Fe)-KF.

The SEM images are shown in Figure 3; most of the crystals in the MIL-100(Fe)-HF sample had the typical MIL-100(Fe) bipyramidal shape, while small round crystals could also be seen. Taking into account the above characterization and literature data, it can be said that the round crystals could be ascribed either to the Fe(BTC) phase or to smaller MIL-100(Fe) crystals that had not yet formed the bipyramids. If we look at images of the samples synthesized without HF or without fluorine, we can see that the number of bipyramidal crystals was smaller, indicating that the presence of fluorine in the synthesis was essential to form MIL-100(Fe), while the absence of HF in the synthesis led to the formation of more unknown phase.

**Figure 4:**
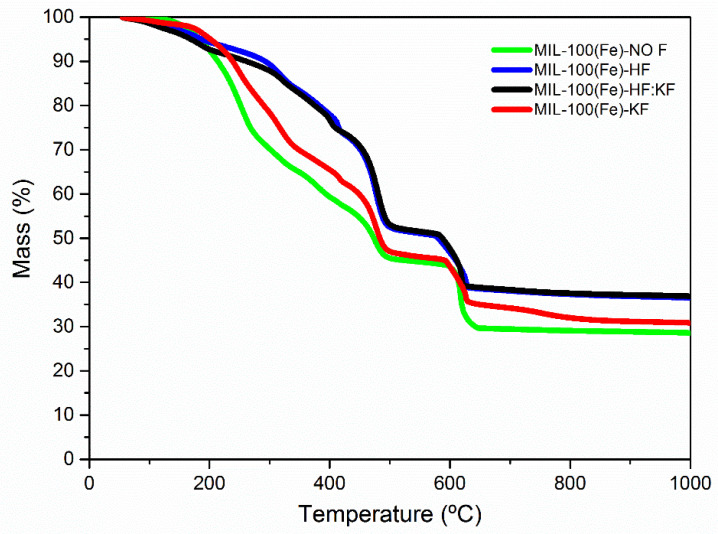
Thermogram of MIL-100(Fe) synthesized.

Figure 4 shows the mass loss profile of the four samples when subjected to a temperature program from room temperature to 1000 °C in an inert gas flow. As can be checked, the MIL-100(Fe)-HF:KF and MIL-100(Fe)-HF samples presented a profile with several weight losses: A first weight loss at 180 °C, another from 220 °C to 380 °C, a third from 380 °C to 540 °C, and the last centered at 620 °C. In addition, both samples had a residual mass of 37 wt.% after treatment. The samples prepared in the absence of HF presented a different profile, with four weight losses, i.e., the ones mentioned above, but with different ratios of weight loss. The residual mass was between 31 wt.% and 28 wt.% for these samples.

**Figure 5:**
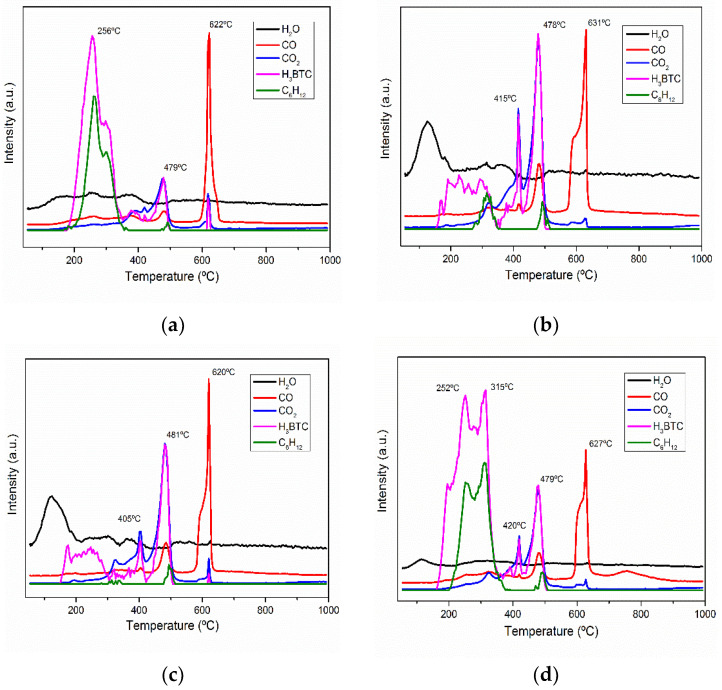
Gases emitted upon thermogravimetry analysis of the samples: (**a**) MIL-100(Fe)-NO F; (**b**) MIL-100(Fe)-HF; (**c**) MIL-100(Fe)-HF:KF; (**d**) MIL-100(Fe)-KF.

To elucidate the cause of each weight loss, the gases emitted during the experiment were measured. If we look at Figure 5 that represents the gases emitted by all sample, we can see that the peak at 180 °C was due to the loss of water, accompanied by a signal of *m*/*z* = 18. It is important to remember that MIL-100(Fe) is a hydrophilic material.

The second weight loss between 220 °C and 380 °C was quite different for the four samples. For MIL-100(Fe)-HF and MIL-100(Fe)-HF:KF, it occurred at temperatures closer to 380 °C, whereas, for MIL-100(Fe)-NO and MIL-100(Fe)-KF, it took place at temperatures closer to 220 °C. This again indicates that the presence of HF generated materials with different properties. If we now analyze the gases emitted in the decomposition of the samples, we can perceive the signals *m*/*z* = 45 and 74, associated with trimesic acid. This suggests that a phase of the sample that is not very stable was decomposed. Sanchez-Sanchez [45] published that the unknown phase Fe(BTC) is less stable than MIL-100(Fe); hence, we can state that this stage was due to the decomposition of the Fe trimesate Fe(BTC). If we focus on the MIL-100(Fe)-NO and MIL-100(Fe)-KF samples, we can see that the amorphous phase of the MIL-100(Fe)-KF sample decomposed at a slightly higher temperature than the MIL-100(Fe)-NO sample. This indicates that the amorphous phase was also stabilized in the presence of fluorine.

If we focus on the third mass loss between 380 and 540 °C, we can appreciate that the masses associated with the linker decomposition showed a maximum in this region. This mass loss could be associated with the decomposition of MIL-100(Fe). As can be noticed, all samples decomposed at the same temperature. The main difference was in the amount of gases emitted and the mass loss, which were higher for the samples prepared in the presence of HF. In the case of samples prepared without HF, it can be seen that the sample with KF also had a higher mass loss.

The last mass loss centered at 620 °C was due to the reduction of the iron oxides formed in the previous stages with the carbon residues. This can also be seen in the gases emitted (CO, CO_2_) and the temperature at which it occurred, 620 °C, which matches very well with the Ellingham diagram [46].

These results indicate that, whatever the preparation method, we obtained an amorphous Fe(BTC) phase that decomposed at a lower temperature and a crystalline MIL-100(Fe) phase that degraded at a higher temperature. Through analyzing the weight loss at both stages, it was possible to quantify how much mass loss was due to the amorphous phase and the crystalline phase, providing a semiquantitative indication of the amount of each phase. However, we cannot discern exactly how much of each phase is present because we do not know the exact composition (molecular weight) of the amorphous phase. From the data found in the literature, we can only say that the amorphous phase is richer in organic component (linker) than the crystalline phase [45]. This indicates that, according to a comparison of the mass loss, we overestimated the amorphous phase, but we do not know by how much. Table 2 shows the ratios of the two phases calculated from the percentage of mass loss.

**Figure A1:**
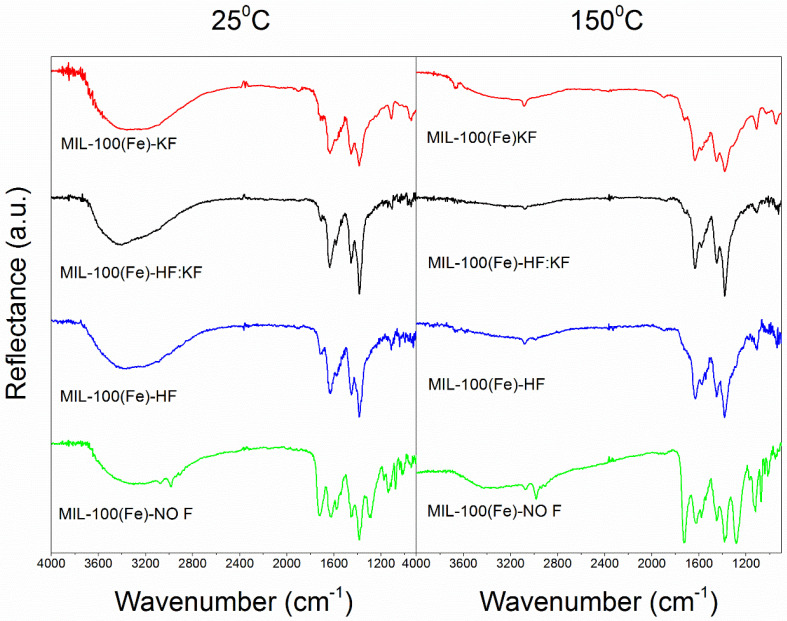
FT-IR spectra of MIL-100(Fe) samples after synthesis and after treatment at 150 °C in inert atmosphere.

In order to analyze the functional groups in the samples, the IR spectra were measured (Figure A1). For this purpose, the samples were measured after being synthesized and after being treated at 150 °C. The spectra show two distinct zones, one below 2000 cm^−1^ and one above 2400 cm^−1^. The zone below 2000 cm^−1^ showed peaks associated with the vibrations of the organic part. The assignment of each peak has been published by several authors [33,44,45,47]. The most remarkable change we detected was the peak developed at 1700 cm^−1^ in the sample MIL-100(Fe)-NO F, described as the stretching vibration of an uncoordinated carboxylate. This is very much in line with what we found using other characterization techniques, i.e., the sample prepared without a fluorine source was more defective.

The peaks located above 2800 cm^−1^ were associated with the vibrational modes of water. This peak is usually broad due to the interactions of the water molecules with each other. This broad peak shows that the samples were hydrophilic, as published in several articles. When the samples were heated, the peak associated with water decreased, being more evident in the MIL-100(Fe)-HF:KF and MIL-100(Fe)-HF samples. This indicates that these two samples were less hydrophilic, suggesting that the number of defects of unsaturated metal centers was lower in these samples, and that they adsorbed less water. These results are related to those obtained using other techniques, as described above.

**Figure A2:**
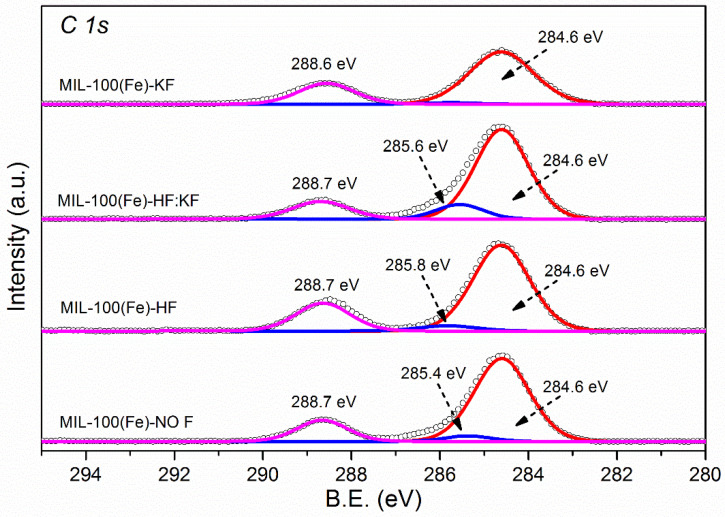
XPS spectra of the C 1s core level for all samples as prepared.

**Figure A3:**
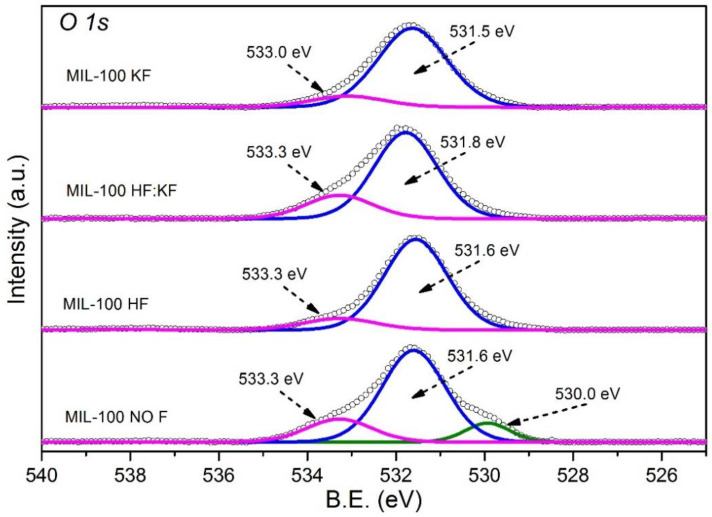
XPS spectra of the O 1s core level for all samples as prepared.

The XPS spectrum of C 1s is shown in Figure A2, which could be deconvoluted into three peaks centered at 284.8, 288.9, and 285.1 eV. The peaks at 284.8 and 288.9 eV corresponded to phenyl and carboxyl carbon signals, respectively. The peak at about 285.1 eV was assigned to carbon on the surface of the sample. There was no significant difference between the different samples. If we now focus on the O 1s spectrum in Figure A3, we can see that the samples prepared in the presence of fluorine showed two well-defined peaks at 533.3 and 531.5 eV, which were ascribed to the different oxygen species coordinated with the metal. However, sample MIL-100(Fe)-NO showed another peak at 530 eV, associated with the carboxylic group oxygen uncoordinated with the metal. This can be explained by the higher number of defects and the presence of the Fe(BTC) phase in this sample. The absence of this peak in the other samples does not imply no defects, but rather that the number of defects was smaller and difficult to detect using XPS.

**Figure 6:**
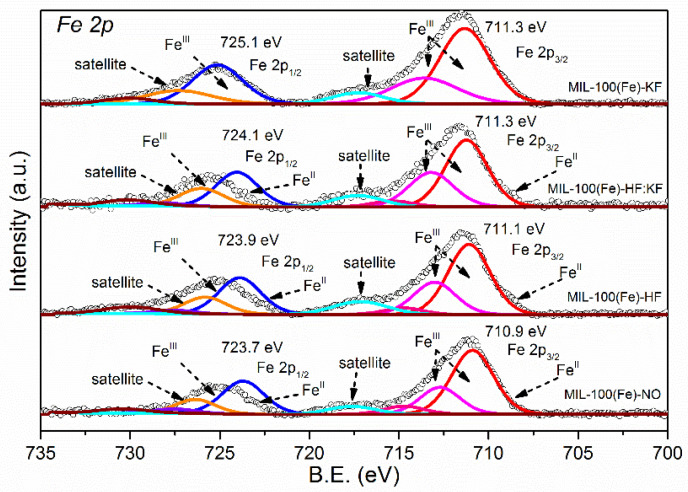
XPS spectra of the synthesized samples. The figure shows the Fe 2p core-level spectra.

The analysis of the Fe 2p spectrum was complex as the Fe(III) spectrum showed satellite peaks in addition to those corresponding to pulling an electron from the 2p orbital. Figure 6 shows the spectra of all the samples. It can be seen that all samples presented a similar spectrum associated with Fe(III). The presence of Fe(II) is difficult to discern as the Fe(II) and Fe(III) peaks have a similar binding energy and cannot always be distinguished. In this particular case, there seemed to be no Fe(II). This is reasonable since the samples were not heat-treated prior to the experiment. Daturi et al. found Fe(II) in MIL-100(Fe) samples, but this was after treating the samples at high temperatures of 200 °C to induce a reduction of Fe(III) to Fe(II) [30].

**Figure 7:**
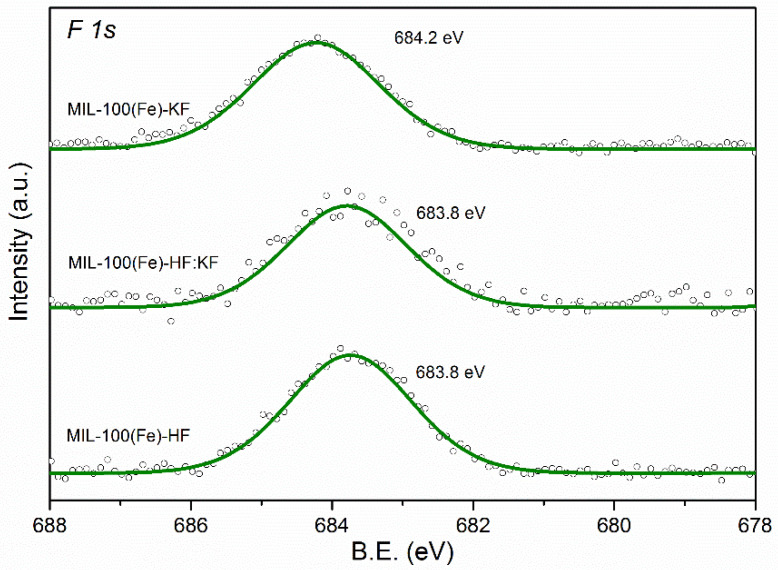
XPS spectra of the synthesized samples. The figure shows the F 1s core-level spectra.

If we now look at the F 1s spectra (Figure 7) of the samples prepared in the presence of fluorine, it can be seen that only one peak appeared, centered at 683.8 eV for samples MIL-100(Fe)-HF:KF and MIL-100(Fe)-HF and at 684.2 eV for sample MIL-100(Fe)-KF. In all cases, this peak was located at a binding energy characteristic of metal fluorides. The difference in binding energy between MIL-100(Fe)-KF and the other two samples was due to the fact that HF was not used in the synthesis, resulting in a more defective sample. Accordingly, the fluorine had to transfer more electron density to the metal cluster to stabilize it, resulting in a higher binding energy. Another important fact that we found using XPS is that the Fe/F ratio was 0.33 (Table A2), i.e., one atom of fluorine for every three atoms of Fe. This corresponds very well to one fluorine atom per metal cluster. This ratio was found in all samples, indicating that, regardless of the fluorine source, the amount of fluorine in the final structure was not altered.

Taken altogether, it can be concluded that, under the synthesis conditions used, two phases were generated: MIL-100(Fe) and the so-called Fe(BTC). The ratio of the two phases differed depending on the fluorine source used. Furthermore, we can see that, when no fluorine was used and the fluorine source was not HF, the samples had a higher proportion of Fe(BTC) and a higher amount of uncoordinated carboxylic groups, thus causing a large number of defects.

As shown above, the prepared samples had very different textural and chemical properties and can be expected to behave differently as catalysts. As mentioned above, MIL-100(Fe) has two functionalities inherent to its structure: Lewis acidity and a redox pair, depending on how the sample is synthesized. Thus, the samples were used as catalysts in the cycloaddition of CO_2_ with epoxide in the presence of a cocatalyst.

**Figure 8:**
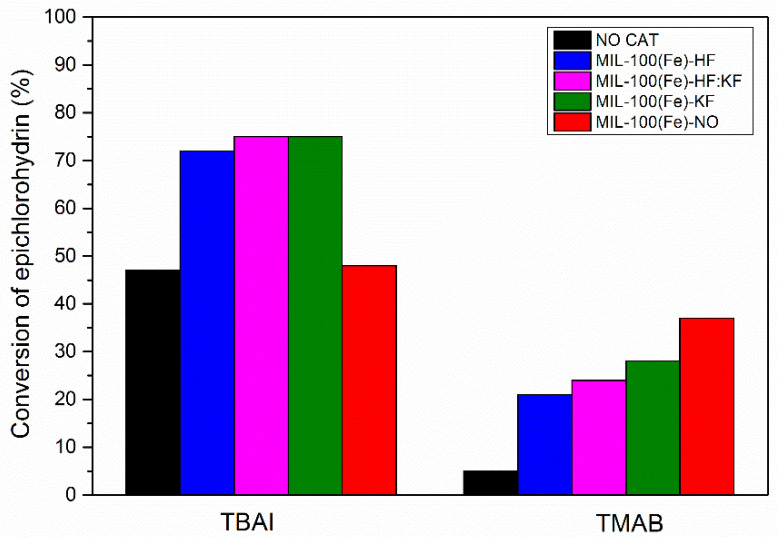
Catalytic reaction of CO_2_ with epichlorohydrin with different MIL-100(Fe) samples as catalysts and organic halides as cocatalysts. Conditions: 97 mol.% of epichloroydrin, 0.84 mol.% of MIL-100(Fe), 1.53 mol.% of organic halide, 0.57 mol.% of mesytilene, 60 °C, 15 bar CO_2_, 250 min [49]. The conversion of epichlorohydrin was based on ^1^H-NMR results (chloropropene carbonate was the only product).

In the first experiments, we used tetrabutylammonium iodide (TBAI) as a cocatalyst (see Figure 8). As can be seen, when only the cocatalyst is used, the reaction proceeded in the absence of MIL-100(Fe). When the catalysts were used, the conversion increased, showing that the prepared samples catalyzed this reaction. However, it was difficult to discern a trend among the catalysts tested, let alone establish a correlation between catalytic activity and sample properties. This was due to two factors: (i) the TBAI catalyzed the reaction by itself to a large extent, hindering the observation of the intrinsic catalytic properties of the samples; (ii) the TBAI was very large, preventing access to the pores, which resulted in catalysis taking place on the surface of the crystals [48].

Following these results, we decided to use tetramethylammonium bromide (TMAB) [50] as a cocatalyst, as it is considerably smaller than TBAI and can penetrate the porosity of the material. When we used this cocatalyst, we can see that the cocatalyst alone did not catalyze the reaction, as the conversion was practically zero (See Figure 8). When the catalysts we prepared were added, the conversion increased in the following order MIL-100(Fe)-NO < MIL-100(Fe)-KF < MIL-100(Fe)-HF:KF < MIL-100(Fe)-HF. This correlates with their properties, as MIL-100(Fe)-NO, despite having less surface area than MIL-100(Fe)-HF, performed better as a catalyst. We attributed this to the higher number of defects in the sample prepared without fluorine, indicating that the active centers of this catalyst were related to the defects [49,51].

Table A2 shows the conversion, selectivity, and reaction conditions when other solids were used as catalysts for CO_2_ cycloaddition, as well as the conditions followed for other mesoporous solids. Entries 1–4 present the studies carried out with SBA-15 and MFI (mesoporous silica), TS-1 (mesoporous zeolite), and MCM-41 (mesoporous material from a family of silicate and alumosilicates). Entries 5–9 show the results from MOFs. ZIF-8, HKUST-1, and MIL-125 were considered microporous MOFs. MIL-101(Cr) and MIL-100(Cr) were treated as mesoporous MOFs. Lastly, entry 10 was the MIL-100(Fe) studied in this work. We also recommend the book chapter published by Campello et al. [52].

**Figure 9:**
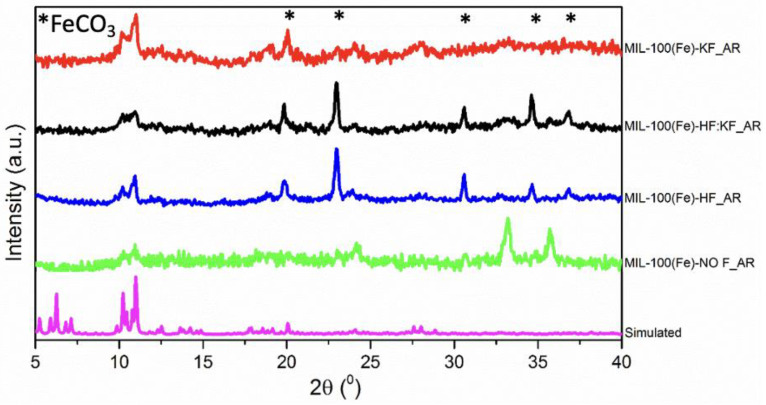
XRD pattern of the MIL-100(Fe) samples after the reaction (AR), compared to the simulated MIL-100(Fe). * Show the FeCO_3_ phase.

Once we verified that the catalysts catalyzed the reaction, it was important to check their stability during the reaction. To do this, we measured the X-ray diffraction of the samples after they were used in the reaction. Figure 9 shows these results. As can be seen, the X-ray diffraction pattern of the used samples was different from the fresh samples in all cases, except for MIL-100(Fe)-NO F, which maintained its structure despite losing crystallinity. It can be noted that a new phase was generated, which was ascribed to iron carbonate (FeCO_3_), indicating that the samples degraded during the reaction. With these results in mind, we also analyzed the reaction medium after the catalytic test using ICP to check for any metal leaching. We found no Fe in the reaction medium; hence, we could rule out that the catalytic activity was due to Fe leaching. In the event that leaching came from the surface, the reaction medium of the MIL-100(Fe) containing fluorine would have featured iron, since the phase in the XRD patterns prior to the reaction was MIL-100(Fe).

**Figure A4:**
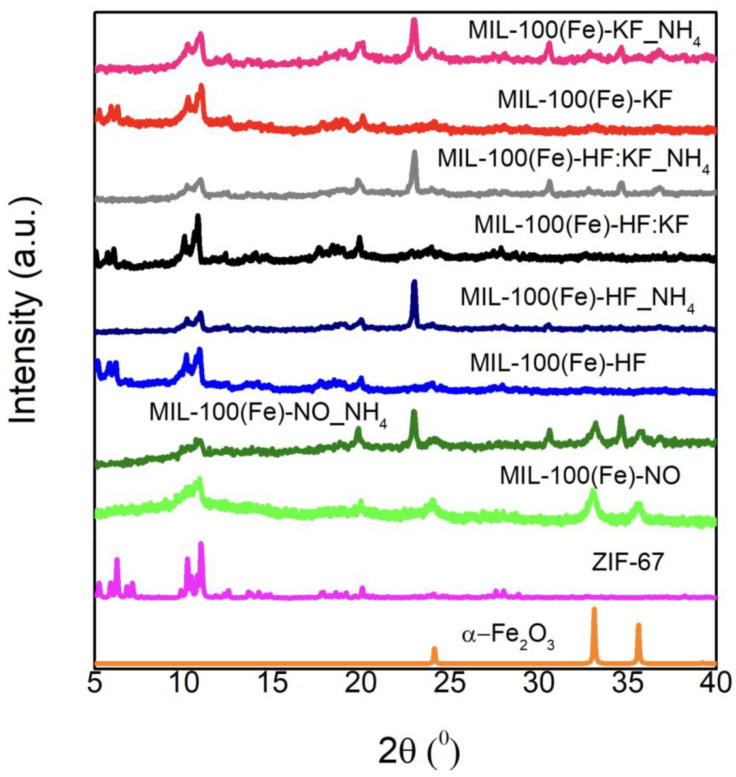
Comparison of the XRD patterns of the samples treated and untreated with NH_4_F.

**Figure A5:**
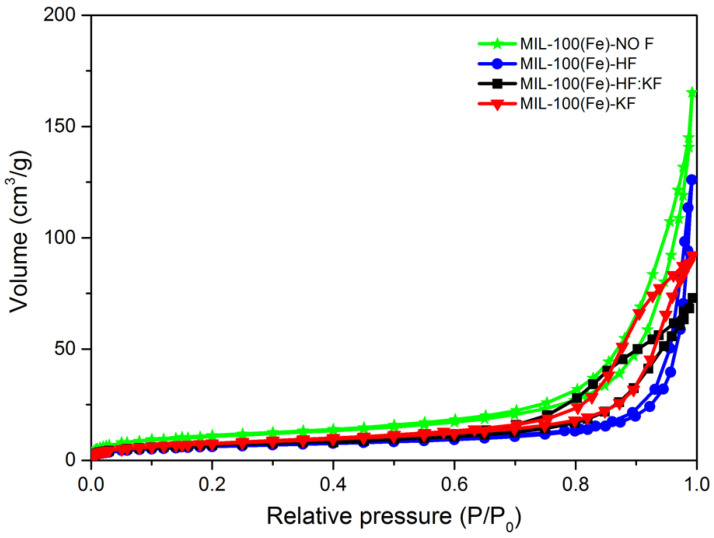
Comparison of the N_2_ adsorption isotherm of the samples treated and untreated with NH_4_F.

Therefore, it is unclear whether the catalytic activity was due to the newly created phase or to MIL-100(Fe). To check this, we intentionally degraded the sample by immersing it in a solution of ammonium fluoride. The X-ray diffractogram of the NH_4_F-treated samples is shown in Figure A4, revealing that, when the samples were treated with NH_4_F, the activity of the iron carbonate phase was very prominent. These samples were characterized by N_2_ adsorption (Figure A5), indicating that the specific surface area decreased considerably. They were also tested in catalysis, showing that their catalytic capacity was much lower than that of the untreated samples. Unfortunately, these results show that the samples were catalytically active, but the catalyst degraded during the reaction.

## 4. Conclusions

The main conclusion that can be drawn from this study is that we can prepare different iron terephthalates (MIL-100(Fe), Fe(BTC)) depending on the fluoride source. Using TGA-MS, we can semiquantitatively determine how much of each phase is found in each sample. If we do not use fluorine in the synthesis or if the source of fluorine is not HF, more defective samples tend to be formed. These more defective samples are more active in the CO_2_ cycloaddition reaction. However, under reaction conditions, the samples are transformed into a more inactive phase.

## Figures and Tables

**Table 1:** Values of specific surface area, micropore volume, and mesopore volume for the synthesized MIL-100(Fe), determined from N_2_ sorption measurements.

Samples	Specific Surface Area (m^2^·g^−1^)	V_micro_ (cm^3^·g^−1^)	V_meso_ (cm^3^·g^−1^)
MIL-100(Fe)-NO F	453	0.24	0.13
MIL-100(Fe)-HF	946	0.45	0.33
MIL-100(Fe)-HF:KF	962	0.46	0.23
MIL-100(Fe)-KF	576	0.32	0.11

**Table 2:** Ratio of the different phases calculated by TGA.

Sample	Weight Loss wt.%	
	Fe(BTC)	MIL-100(Fe)
MIL-100(Fe)-NO	64	36
MIL-100(Fe)-HF	31	69
MIL-100(Fe)-HF:KF	32	68
MIL-100(Fe)-KF	55	45

## Appendix A

**Table A1:** Main characteristic peaks of the synthesized samples MIL-100(Fe) obtained using PDRX. The data are compared with results obtained by Horcajada [20] and Blake [53].

Phase	h,k,l	2θ (°)	d-Spacing	d_NO F_	d_HF_	d_HF:KF_	d_KF_
MIL-100	4,2,2	5.90	14.97	-	15.07	15.33	-
	5,1,1	6.26	14.11	-	14.22	14.55	14.27
	8,2,2	10.22	8.64	-	8.70	8.79	8.72
	9,1,1	10.98	8.05	8.10	8.10	8.18	8.09
	15,5,5	20.06	4.42	4.44	4.43	4.45	4.43
	1,0,−2	24.12	3.69	3.70	-	-	3.69
	1,0,4	33.12	2.70	2.71	-	-	2.71
	2,−1,0	35.62	2.51	2.52	-	-	2.52
α-Fe_2_O_3_	1,0,−2	24.12	3.69	3.70	-	-	3.69
	1,0,4	33.12	2.70	2.71	-	-	2.71
	2,−1,0	35.62	2.51	2.52	-	-	2.52

**Table A2:** Comparison of catalytic performance of reported mesoporous materials in the fixation of CO_2_ in epichlorohydrin.

Entry	Catalyst	Ep./Catalyst (mmol/mg)	Conditions	Solvent	Conv. (%)	Sel. (%)	Ref.
1	SBA-15	18/100	4 h, 120 °C, 6.9 bar	Acetonitrile	1.5	9.0	[54]
2	MFI	10/100	4 h, 140 °C, 20 bar	No solvent	90.1	95.0	[55]
3	TS-1	18/100	4 h, 120 °C, 6.9bar	Methylene chloride	70.8	73.0	[56]
4	MCM-41	18/50	3 h, 120 °C, 6.9 bar	Acetonitrile	99.0	80.0	[57]
5	ZIF-8	18/100	4 h, 80 °C, 7 bar	No solvent	84.0	52.0	[58]
6	HKUST-1	18/100	4 h, 100 °C, 7 bar	No solvent	64.0	52.0	[59]
7	MIL-125	5/20	6 h, 100 °C, 20 bar	No solvent	64.0	99.0	[60]
8	MIL-101(Cr)	9.2/57	24 h, 35 °C, 1.5 bar	No solvent	99.0	99.0	[61]
9	MIL-100(Cr)	20/25	12 h,60 °C, 10 bar	No solvent	11.0	99.0	[62]
10	MIL-100(Fe) ^1^	203/1627	4 h, 60 °C, 15 bar	No solvent	21.0	99.0	This work

^1^ The chosen reaction was carried out with MIL-100(Fe)-HF since this is the standard reaction found in literature. On the other hand, the results obtained with TMAB cocatalyst were chosen due to its smaller size than TBAI, thereby minimizing internal diffusion problems.
